# Exploring psychological symptoms and associated factors in patients receiving medication-assisted treatment for opioid-use disorder

**DOI:** 10.1192/bjo.2019.99

**Published:** 2020-01-08

**Authors:** Tea Rosic, Andrew Worster, Lehana Thabane, David C. Marsh, Zainab Samaan

**Affiliations:** Department of Psychiatry and Behavioral Neurosciences, McMaster University; and Department of Health Research Methods, Evidence, and Impact, McMaster University, Canada; Department of Health Research Methods, Evidence, and Impact, McMaster University; and Department of Medicine, McMaster University, Canada; Department of Health Research Methods, Evidence, and Impact, McMaster University; Department of Medicine, McMaster University; Departments of Pediatrics/Anesthesia, McMaster University; and Biostatistics Unit, Research Institute at St Joseph's Healthcare, Canada; Northern Ontario School of Medicine; and Canadian Addiction Treatment Centres, Canada; Department of Psychiatry and Behavioral Neurosciences, McMaster University; and Department of Medicine, McMaster University, Canada

**Keywords:** Comorbidity, methadone, buprenorphine, addiction, psychiatric symptoms

## Abstract

**Background:**

Patients receiving treatment for opioid-use disorder (OUD) may experience psychological symptoms without meeting full criteria for psychiatric disorders. The impact of these symptoms on treatment outcomes is unclear.

**Aims:**

To determine the prevalence of psychological symptoms in a cohort of individuals receiving medication-assisted treatment for OUD and explore their association with patient characteristics and outcomes in treatment.

**Method:**

Data were collected from 2788 participants receiving ongoing treatment for OUD recruited in two Canadian prospective cohort studies. The Maudsley Addiction Profile psychological symptoms subscale was administered to all participants via face-to-face interviews. A subset of participants (*n* = 666) also received assessment for psychiatric disorders with the Mini International Neuropsychiatric Interview. We used linear regression analysis to explore factors associated with psychological symptom score.

**Results:**

The mean psychological symptom score was 12.6/40 (s.d. = 9.2). Participants with psychiatric comorbidity had higher scores than those without (mean 16.8 *v*. 8.6, *P*<0.001) and 31% of those with psychiatric comorbidity reported suicidal ideation. Higher psychological symptom score was associated with female gender (*B* = 1.59, 95% CI 0.92–2.25, *P*<0.001), antidepressant prescription (*B* = 4.35, 95% CI 3.61–5.09, *P*<0.001), percentage of opioid-positive urine screens (*B* = 0.02, 95% CI 0.01–0.03, *P*<0.001), and use of non-opioid substances (*B* = 1.92, 95% CI 0.89–2.95, *P*<0.001). Marriage and employment were associated with lower psychological symptoms.

**Conclusions:**

Psychological symptoms are associated with treatment outcomes in this population and the prevalence of suicidal ideation is an area of concern. Our findings highlight the ongoing need to optimise integrated mental health and addictions services for patients with OUD.

## Background

In recent years, public and scientific attention surrounding addictions has been dominated by the ‘opioid epidemic’, which remains an ongoing public health crisis across North America and increasingly in Europe.^1^ High rates of opioid use, opioid-use disorder (OUD) and opioid-related deaths persist despite efforts to reduce prescribing of opioid medications and increase available treatments and harm-reduction strategies. The rise in opioid-related deaths, increasing by more than 20% on average between 2011 and 2016, has been most pronounced in the USA, Canada, Sweden, Norway, Ireland, and England and Wales.^1^ Medication-assisted treatment (MAT) for OUD, including methadone, a full opioid agonist and buprenorphine, a partial opioid agonist, have demonstrated benefits in the reduction of prescription and non-prescription opioid use;^2,3^ however, not all patients have favourable outcomes in treatment.^4^ Individuals with OUD have a high prevalence of comorbid psychiatric disorders including depression and anxiety.^5,6^ These comorbidities have been associated with worse retention in treatment,^7^ opioid use^5,6^ and mortality.^8^ Many patients may also experience psychological symptoms that do not meet full diagnostic criteria for psychiatric disorders but may compromise well-being or impair function nonetheless.^7^

The problem of psychological symptoms in patients receiving treatment for substance use disorders is one of significant complexity. It has long been acknowledged that individuals with substance use disorders have a high prevalence of other comorbid psychiatric disorders.^9,10^ Psychological symptoms may also be ‘substance-induced’ and occur secondary to a primary substance use disorder, or in the context of intoxication or withdrawal.^11,12^ Numerous theories exist to explain the high rate of comorbidity including common underlying genetic predisposition, common neurobiological pathways and diagnostic confounding.^12,13^ The natural history of psychological symptoms during MAT, and the implications for treatment outcomes, are not definitively understood. Unfortunately, patients with psychiatric comorbidity are often excluded from experimental studies of MAT for OUD on the basis of their comorbidity.^14^ Therefore, observational studies must be relied upon to obtain information on these patients and their course in treatment. There is an enduring need to optimise the integration of mental health and addictions services; parallel, rather than integrated, treatment continues to be the dominant conceptual framework and therefore the dominant approach to care.^15^ Resources available in addictions treatment to address psychological or psychiatric comorbidity are often limited.^15^ A better understanding of the psychological symptoms experienced by patients with OUD has the potential to inform clinical assessments, management of underlying comorbidity, treatment decision-making and resource allocation.

## Objectives

The objectives of this study are as follows: (a) to determine the prevalence of psychological symptoms in a cohort of patients receiving MAT for OUD; (b) to determine the prevalence of psychological symptoms among patients in this cohort with known comorbid psychiatric disorders versus those without psychiatric comorbidity; and (c) to explore the association between psychological symptoms and demographic and clinical characteristics, including treatment outcomes such as ongoing opioid and non-opioid substance use.

## Method

### Data

We used data from two large, prospective, cohort studies: the GENetics of Opioid Addiction (GENOA) study and the Pharmacogenetics of Opioid Substitution Treatment Response (POST) study. Study methods for the GENOA project have been previously described.^5,16^ Briefly, data collection for the GENOA cohort occurred between 2011 and 2017, and information is available on 1390 individuals receiving treatment for OUD across 20 out-patient MAT clinics within Ontario, Canada. Participants were eligible for study inclusion if they were at least 18 years old, diagnosed with OUD as per the DSM-IV criteria,^17^ and enrolled in MAT for their OUD. At study entry, participants provided information on demographic characteristics, medical history, medications and clinical information on their MAT medication, dose and duration in treatment. The POST study began recruitment in May 2018 from out-patient MAT clinics within Ontario, Canada and 1769 participants were recruited as of June 2019. Inclusion criteria for this study were similar to that of the GENOA study: individuals diagnosed with OUD as per the DSM-5 criteria^18^ and receiving MAT. Participants completed a similar baseline assessment including demographic and clinical information. Participants recruited to both studies were enrolled in ongoing MAT, for varying lengths of time, at the time of study recruitment. In both studies, participants were followed for 12 months and urine drug screens to assess for ongoing opioid or other substance use were conducted as per clinical protocol. All clinical sites included in the studies are run centrally by the same management teams through the Canadian Addiction Treatment Centres and all follow the same treatment protocols. The authors assert that all procedures contributing to this work comply with the ethical standards of the relevant national and institutional committees on human experimentation and with the Helsinki Declaration of 1975, as revised in 2008. Ethics approval was obtained from the Hamilton Integrated Research Ethics Board (GENOA project ID 11-056; POST project ID 4556), and all participants provided verbal and written informed consent.

**Fig. 1:**
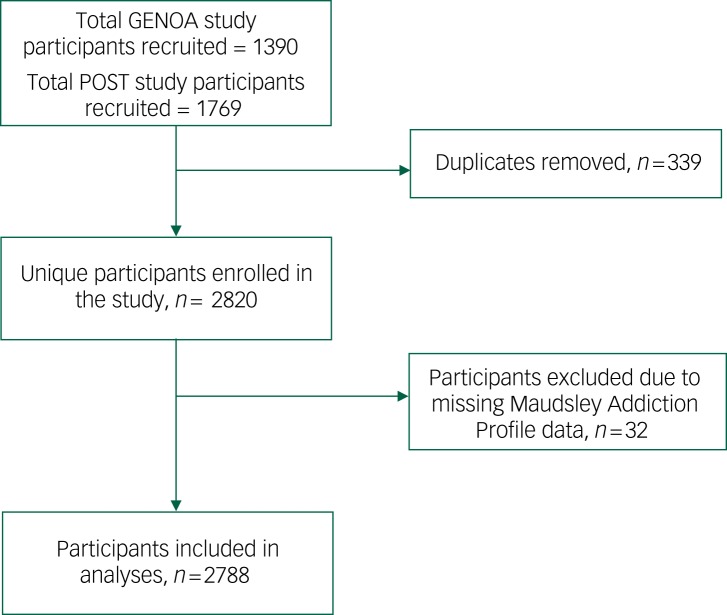
Study flow diagram.

GENOA, GENetics of Opioid Addiction; POST, the Pharmacogenetics of Opioid Substitution Treatment Response study.

Records from these two cohort studies were merged and duplicate enrolment was identified using patient name and birth date. In the event of duplicate enrolment, data collected in the GENOA study were retained and the duplicate from the POST study was removed. Duplicate enrolment in both studies affected 339 participants (Fig. 1, study flow diagram). Participants were excluded from this study's main analyses if they were missing baseline data on psychological symptoms (Fig. 1). This study is reported in accordance with the Strengthening the Reporting of Observational Studies in Epidemiology (STROBE) guidelines.^19^

### Study instruments and measures

The primary objective of this study was to determine the prevalence of psychological symptoms in patients receiving MAT for OUD. At study entry all participants were administered the Maudsley Addiction Profile (MAP),^20^ a validated questionnaire examining individuals’ self-reported substance use, physical and psychological symptoms and social functioning in the past 30 days. Psychological symptoms of depression and anxiety are measured by ten items rated on five-point Likert scales and participants are asked to report the frequency of symptoms including tension, fear, nervousness, panic, hopelessness, worthlessness, anhedonia, loneliness and suicidal ideation they have experienced in the past month.^20^ Each item is rated as occurring ‘never’, ‘rarely’, ‘sometimes’, ‘often’, or ‘always’.^20^ A total score for psychological symptoms is calculated by adding the item scores for a total symptom score that can range from 0 to 40.^20^ The psychological symptoms subscale is derived from the Brief Symptom Inventory,^21^ which is derived from the Symptom Check List 90.^22^ All participants received face-to-face interviews with a research assistant and the data were entered into the Research Electronic Data Capture tool.^23,24^ The first 666 participants consecutively recruited into the GENOA study received extensive assessment of psychiatric comorbidity using the MINI International Neuropsychiatric Interview version 6.0.^25^ This allowed for identification of comorbid psychiatric disorders including mood disorders, anxiety disorders, psychotic disorders, substance use disorders and eating disorders. The MINI was not administered to subsequently recruited participants because of the time burden of administration.

Assessment of treatment outcome was conducted using urine drug screen results to identify continued opioid use. An opioid-positive urine screen was defined as a non-methadone- or buprenorphine-positive opioid screen. We were therefore able to calculate the percentage of opioid-positive urine screens for each participant. As participants report on psychological symptoms in the past 30 days at time of study entry, we elected to examine urine drug screens also in the period prior to study entry. By doing so, the results of both psychological symptom assessment and urine drug screens were as close in time as possible. In the GENOA study, the results of urine drug screens were available for the 3-month interval prior to study entry and the mean number of urine drug screens was 16. In the POST study, the results of urine drug screens were available for up to 12 months prior to study entry and the mean number of urine drug screens was 45. To further assess treatment outcome, we also examined the percentage of non-opioid-positive urine drug screens collected during the same period prior to study entry. This includes urine screens positive for amphetamines, benzodiazepines and cocaine. Amphetamine- or benzodiazepine-positive urines were not counted for patients with confirmed amphetamine or benzodiazepine prescriptions in their medical chart. Urine drug screens were conducted as per routine clinical care at the Canadian Addiction Treatment Centre sites, at a weekly or fortnightly frequency, using the IMDx^TM^ Prep assay^26^ for morphine, oxycodone, fentanyl, cocaine, amphetamine, methamphetamine, diazepam, methadone metabolite and buprenorphine.

### Analysis

Our first objective was to determine the prevalence of psychological symptoms in this study cohort. We used Stata version 15.1^27^ to conduct all statistical analyses. We present data on demographic characteristics and psychological symptoms using descriptive statistics with continuous variables summarised as means and s.d. for normally distributed variables, and medians with interquartile range (IQR) for skewed data. Categorical variables were summarised as percentages. Our second objective was to assess the prevalence of psychological symptoms among patients with known comorbid psychiatric disorders identified using the MINI, who we compared with those patients with no psychiatric comorbidity identified. Group differences were assessed using independent samples *t*-tests for continuous variables and chi-square tests for dichotomous variables. Our third and final objective was to explore the association between participant characteristics and psychological symptoms.

We constructed a linear regression model with total psychological symptom score (ranging from 0 to 40) as the dependent variable. Sociodemographic characteristics included as covariates in the regression analysis were selected based on previous research suggesting potential influence on psychological symptoms. These included: (a) age, as older age has been associated with underreporting of psychological symptoms;^28^ (b) gender, because of known gender differences in mental health disorders and psychological patterns such as internalising and externalising;^29^ (c) marital status, which has been demonstrated to confer mental health benefits for both men and women;^30^ and (d) employment status, as unemployment has been established in numerous studies to impair mental health.^31^

The clinical characteristics included as covariates in the regression model included: (a) type of MAT (i.e. methadone or buprenorphine), (b) duration in treatment, and (c) prescription of an antidepressant medication. Finally, we included the percentage of opioid-positive urine drug screens (continuous), and the presence of non-opioid substance use (dichotomous) as covariates in the model in order to assess the association between psychological symptoms and substance use during treatment, adjusting for the other aforementioned factors.

The linear regression model was constructed as described above. Model diagnostics were assessed to ensure that the assumptions for linear regression analysis were adequately met. This included testing for multicollinearity with variance inflation factor, testing for homoscedasticity by plotting residuals against fitted values, graphing residuals against a normal curve and assessing P-P and Q-Q plots for assessment of the normality of residuals. The level of significance for hypothesis testing was set at *α* = 0.05 for all analyses. Our sample size of 2788 participants was sufficiently powered to conduct the analyses described above, such that there were at least ten participants per covariate included in the model.^32^ Finally, we conducted subgroup analysis by gender in light of increasing awareness that gender is associated with biological and social differences that contribute to differential health outcomes, which warrant understanding.

## Results

### Demographic and clinical characteristics

Altogether, 2788 participants met study inclusion criteria and were included in the analyses (Fig. 1). Information on participant demographic and clinical characteristics is detailed in Table 1: men accounted for 57% of the cohort (*n* = 1575), and participants’ mean age was 38.5 years (s.d. = 11). The majority of participants held at least a high school diploma (*n* = 2098; 75.3%) but were unemployed (*n* = 1819; 65.2%). Methadone was the most commonly prescribed MAT (85%) and the median methadone dose was 65 mg per day (IQR = 60), and the median dose of buprenorphine was 12 mg per day (IQR = 8). In addition to their MAT medication, 26.4% of participants were prescribed an antidepressant medication (*n* = 737). The median duration in treatment was 2 years (IQR = 4.42). In total, 37% of participants were abstinent from any ongoing opioid use, as measured by urine drug screens, for the 3 months prior to study entry. Individuals with ongoing opioid use had on average 30% of urine screens positive for opioids (s.d. = 29.3). Only 11% of participants were entirely abstinent from non-opioid substances (i.e., amphetamines, benzodiazepines and cocaine).

**Table 1:** Participant demographic and clinical characteristics (*N* = 2788).

Characteristic	Value
*Sociodemographic*	
Age, years: mean (s.d.)	38.5 (11)
Male gender, *n* (%)	1575 (56.5)
White ethnicity, *n* (%)	2207 (79.2)
Married or common law, *n* (%)	840 (30.1)
Education, *n* (%)	
Less than high school	690 (24.8)
High school	1295 (46.5)
Post-secondary	803 (28.8)
Unemployment, *n* (%)	1819 (65.2)
*Clinical*	
Type of medication-assisted treatment, *n* (%)	
Methadone	2369 (85.0)
Buprenorphine	419 (15.0)
Dose, mg/day: median (IQR)	
Methadone	65 (60)
Buprenorphine	12 (8)
Duration in treatment, years: median (IQR)	2 (4.42)
Number of opioid urine drug screens, mean (s.d.)	31.3 (24.2)
Opioid abstinence, *n* (%)	1031 (37.0)
Percentage of opioid-positive urine drug screens among non-abstainers; mean (s.d.)	30.1 (29.3)
Abstinence from other substances, *n* (%)	317 (11.4)
Percentage of positive urine drug screens among non-abstainers of other substances, mean (s.d.)	
Amphetamines (*n* = 229)	57.2 (35.4)
Benzodiazepines (*n* = 559)	32.1 (29.6)
Cocaine (*n* = 935)	49.2 (34.7)
Antidepressant medication prescription, *n* (%)	737 (26.4)

### Psychological symptoms

In the entire study sample of 2788 participants, the mean total psychological symptoms score was 12.6 out of 40 (s.d. = 9.2; Table 2). Altogether, 92% of participants endorsed at least one psychological symptom present in the past 30 days (data not shown). The most commonly endorsed symptoms were feeling tense (80%), feeling no interest in things (70%), feeling lonely (66%) and feeling hopeless about the future (64%; Table 2). The mean total psychological symptoms score was lower in men compared with females (11.4 (s.d. = 8.7) *v*. 14.1 (s.d. = 9.5); *t* = −7.5, *P*<0.001; data not shown).

**Table 2:** Psychological symptoms (*N* = 2788)

Psychological symptoms^a^	Total sample (*N* = 2788)	Participants assessed for comorbid psychiatric disorders^b^ (*n* = 666)				
		Psychiatric comorbidity^c^ (*n* = 400)	No psychiatric comorbidity^d^ (*n* = 266)	χ^2^	*t*-test	*P*
Total score; mean (s.d.)	12.6 (9.2)	16.8 (8.9)	8.6 (6.9)	–	−12.79	<0.001
Feeling tense, *n* (%)	2230 (80.0)	372 (93.0)	206 (77.4)	85.8	–	<0.001
Suddenly scared for no reason, *n* (%)	1187 (42.6)	230 (57.5)	85 (32.0)	62.9	–	<0.001
Feeling fearful, *n* (%)	1304 (46.8)	252 (63.0)	98 (36.8)	72.7	–	<0.001
Nervousness or shakiness inside, *n* (%)	1741 (62.4)	320 (80.0)	139 (52.3)	92.5	–	<0.001
Spells of terror or panic, *n* (%)	1224 (43.9)	251 (62.8)	78 (29.3)	84.9	–	<0.001
Feeling hopeless about the future, *n* (%)	1795 (64.4)	328 (82.0)	148 (55.6)	85.4	–	<0.001
Feelings of worthlessness; *n* (%)	1580 (56.7)	292 (73.0)	115 (43.2)	83	–	<0.001
Feeling no interest in things, *n* (%)	1942 (69.7)	332 (83.0)	158 (59.4)	60.6	–	<0.001
Feeling lonely, *n* (%)	1825 (65.5)	323 (80.8)	144 (54.0)	77.2	–	<0.001
Thoughts of ending your life, *n* (%)	594 (21.3)	123 (31.0)	30 (11.3)	35.4	–	<0.001

a. Each item in the Maudsley Addiction Profile psychological symptoms subscale is assessed on a five-point Likert scale ranging from ‘never’ (score of 0) to ‘always’ (score of 4) experiencing the symptom. In this table we present the frequency of scores of >1 for each symptom, indicating the presence of the symptom but not the intensity.

b. A subset of participants received the Mini International Neuropsychiatric Interview (MINI) diagnostic interview to identify comorbid psychiatric disorders (*n* = 666).

c. Participants identified as meeting criteria for a comorbid mood, anxiety, psychotic or eating disorders using the MINI.

d. Participants identified as not meeting criteria for a comorbid mood, anxiety, psychotic or eating disorders using the MINI.

Among the subset of 666 participants who received comprehensive assessment of psychiatric comorbidity using the MINI, 400 individuals (60%) were identified to have a comorbid mood, anxiety, psychotic or eating disorders. We compared the prevalence of psychological symptoms in these participants, with the prevalence of psychological symptoms in participants identified not to have a psychiatric comorbidity (Table 2). The prevalence of every symptom was higher among those patients with psychiatric comorbidity (Table 2). The total psychological symptoms score was also significantly higher in patients with psychiatric comorbidity than those without (16.8 *v*. 8.6, *P*<0.001; Cohen's *d* = −1.01, 95% CI −1.18 to −0.85). Notably, 31% of participants with psychiatric comorbidity endorsed thoughts of ending their life in the past 30 days, whereas this figure was 11% in participants who were not identified to have psychiatric comorbidity (*P*<0.001). Overall, the prevalence of suicidal ideation was similar in men and women (22% *v*. 20%, χ^2^ = 1.68, *P* = 0.195; data not shown)

### Factors associated with psychological symptom score

Higher psychological symptom score was associated with female gender, antidepressant prescription, ongoing opioid use and use of non-opioid substances (Table 3). Female gender was associated with two points higher on the psychological symptom score, on average, adjusting for the other covariates (*B* = 1.59, 95% CI 0.92–2.25, *P*<0.001). For each percentage point more of opioid-positive urine drug screens, psychological symptom score was associated with a 0.02 increase (*B* = 0.02, 95% CI 0.01–0.03, *P*<0.001). Any use of non-opioid substances detected by urine drug screens was associated with about two points higher psychological symptom score, on average (*B* = 1.92, 95% CI 0.89–2.95, *P*<0.001). Lower psychological symptom score was associated with younger age, (*B* = −0.08, 95% CI −0.11 to −0.05, *P*<0.001), being married or in a common-law relationship (*B* = −1.72, 95% CI −2.43 to −1.02, *P*<0.001), being employed (*B* = −2.75, 95% CI −3.45 to −2.05, *P*<0.001) and treatment with buprenorphine (*B* = −1.13, 95% CI −2.06 to −0.02, *P* = 0.017; Table 3).

**Table 3:** Multivariable model of demographic and clinical factors associated with psychological symptom score (*N* = 2778)

Covariate	Primary analysis			Subgroup analysis					
	Total sample			Women			Men		
	*B*^a^	95% CI	*P*	*B*^a^	95% CI	*P*	*B*^a^	95% CI	*P*
Age, years	−0.08	−0.11 to −0.05	<0.001	−0.08	−0.14 to −0.03	0.002	−0.07	−0.11 to −0.03	<0.001
Female gender	1.59	0.92 to 2.25	<0.001	−	−	−	−	−	−
Married	−1.72	−2.43 to −1.02	<0.001	−2.84	−4.03 to −1.65	<0.001	−1.25	−2.17 to −0.34	<0.001
Employed	−2.75	−3.45 to −2.05	<0.001	−2.24	−3.36 to −1.13	<0.001	−2.72	−3.58 to −1.86	<0.001
Buprenorphine	−1.13	−2.06 to −0.02	0.017	−1.45	−2.93 to 0.03	0.054	−0.89	−2.08 to 0.30	0.141
Duration in treatment, years	0.06	−0.02 to 0.13	0.149	0.11	−0.01 to 0.23	0.072	0.003	−0.10 to 0.11	0.953
Antidepressant Prescription	4.35	3.61 to 5.09	<0.001	4.08	2.98 to 5.18	<0.001	4.61	3.60 to 5.62	<0.001
Percentage of opioid-positive urine screens	0.02	0.01 to 0.03	<0.001	0.04	0.02 to 0.06	<0.001	0.01	−0.003 to 0.03	0.134
Use of non-opioid substances	1.92	0.89 to 2.95	<0.001	1.84	0.19 to 3.49	0.029	1.98	0.67 to 3.29	0.003

a. Estimated beta coefficient.

Subgroup analysis by gender resulted in similar findings to those of the primary analysis described above (Table 3). For both men and women, psychological symptom score was associated with age, marital status, employment, antidepressant prescription and use of non-opioid substances (Table 3). The association between higher psychological symptom score and more ongoing opioid use appears to be present in women (*B* = 0.04, 95% CI 0.02 to 0.06, *P*<0.001) but not in men (*B* = 0.01 95% CI −0.003 to 0.03, *P* = 0.134; Table 3). Treatment with buprenorphine as compared with methadone was not associated with psychological symptom score in men (*B* = −0.89, 95% CI −2.08 to 0.30, *P* = 0.141), but showed a trend towards statistical significance in women (*B* = −1.45, 95% CI −2.93 to 0.03, *P* = 0.054; Table 3).

## Discussion

### Main findings

In this large study involving two cohorts of patients receiving out-patient MAT for OUD, we found that the majority of participants reported psychological symptoms during treatment. Higher psychological symptoms were associated with worse treatment outcomes including more opioid use in women and use of non-opioid substances in both men and women. Our results highlight psychological symptoms as an area of need for patients enrolled in this treatment. These data contribute to a growing body of literature on the mental health of patients with OUD.

### Comparison with findings from other studies

A challenge for situating our findings in the pre-existing literature is the considerable variation between studies in the measures used for the assessment of psychological symptoms. A systematic review by Fingleton *et al* in 2015 examined changes in mental health during MAT and identified 19 different instruments used to measure psychological symptoms in the included studies.^33^ This review concluded that mental health significantly improved in 14 out of 22 included studies though improvements were not always sustained.^31^

There are a handful of previous studies that examined psychological symptoms using the MAP with which our results can be compared.^34–37^ In a small observational study of 15 patients beginning MAT for OUD, psychological symptoms were measured at baseline and following 8 weeks of treatment.^37^ The authors found that the mean psychological symptoms score at baseline was 20.1, and at 8-week follow-up this decreased to 13.^34^ These findings are consistent with ours, seeing as patients in our study were enrolled in treatment for significantly longer than 8 weeks on average (and had a mean psychological symptom score of 12.6 out of 40). The most commonly reported symptoms were ‘feeling tense’ and ‘feeling no interest in things’;^34^ the same was the case in our study. We caution careful interpretation of specific items being more commonly reported as there is a possibility that the individual items reported more frequently reflect a property of the questionnaire itself, rather than symptoms experienced by patients, if these items are worded in a way that makes them more likely to be endorsed. Another observational study including 404 participants found a slightly lower prevalence of symptoms of anxiety and depression than those identified in our cohort.^35^ For example, for the two most commonly reported symptoms, ‘feeling tense’ and ‘feeling no interest in things’, rates in the study were 62–66% and 50–60% at study intake, respectively.

Finally, two randomised controlled trials also used the MAP to assess change in psychological symptoms during treatment.^36,37^ The first study included 235 participants and found the mean psychological symptoms score at baseline to be 29.7 (s.d. = 7.7) with a reduction to 24 after 36 months of treatment.^37^ The second randomised controlled trial also identified ‘feeling tense’ and ‘feeling no interest in things’ as the most commonly reported psychological symptoms at baseline in a cohort of 45 patients receiving methadone treatment in a community setting.^36^ This study also identified a slightly lower prevalence of these symptoms than that in our study: 58 and 56%, respectively, with decreases in prevalence to 53 and 54%, respectively, after 1 year of treatment.^36^

### Interpretation of our findings

What level of psychological symptoms, as measured using the MAP, is clinically significant in terms of having an impact on functioning and requiring intervention is unknown. There are no defined standards for interpretation of the MAP or validation against clinical diagnosis of anxiety or depressive disorders. Our finding that individuals with confirmed psychiatric diagnoses had significantly higher psychological symptom scores than individuals without psychiatric comorbidity supports the suggestion that higher symptoms may have a clinical relevance. This finding suggests that intervention, whether psychological or pharmacological, may be warranted and raises questions about the services provided for managing psychological or psychiatric symptoms in MAT clinics. We also found an association between antidepressant prescription and increased psychological symptoms, suggesting that antidepressant treatment alone is insufficient to eliminate psychological symptoms, although it is certainly possible that without antidepressant treatment these individuals would have even higher psychological symptoms.

#### Suicidal ideation

A troubling finding of this study was that 21% of participants reported suicidal thinking in the past 30 days. This rate was higher among participants with identified psychiatric comorbidity at 31%. Our findings are consistent with previous studies in patients with OUD that have documented suicidal ideation in about 20–25% of patients.^35,36^ In comparison, a study of patients using substances in primary care (most with non-opioid substance use) found a 12.1% prevalence of past month suicidal ideation.^38^ Suicidal ideation is a significant concern in patients with OUD, a population in which rates of overdose and mortality are alarmingly high and increasing.^1^ Recent US estimates indicate that more than 40% of suicide and overdose deaths in 2017 involved opioids^39^ and rates of suicide by opioid overdose are likely underestimated as it is difficult to assess intent in overdose deaths.^40^ Opioids have high lethality in overdose, whether unintentional or intentional and there is strong evidence that access to lethal means increases risk for suicide.^41^ This points to a pressing need to identify and manage suicidal ideation in this high-risk population.

#### Buprenorphine versus methadone

We found that treatment with buprenorphine was associated with lower psychological symptoms, as compared with treatment with methadone, although this finding did not hold in subgroup analysis by gender. There is no readily discernible biological explanation for this finding and its significance is unclear. Notably, Fingleton *et al* identified tentative evidence to suggest methadone was less effective at improving mental health than other types of MAT.^33^ It is possible that there are systematic differences between patients who end up on treatment with buprenorphine as compared with methadone that could explain the finding that they have fewer psychological symptoms. We examined differences in demographic characteristics in our participants with methadone compared with buprenorphine treatment and found no significant differences between the groups in age (mean age 38.6 *v*. 37.7, *P* = 0.133), gender (57% *v*. 56%, *P* = 0.876), high school education (47% *v*. 44%, *P* = 0.259) or marital status (30% *v*. 32%, *P* = 0.312). In contrast, there was a significant difference between the two treatment groups in mean duration in treatment (methadone 4.2 years *v*. buprenorphine 1.8 years, *P*<0.001) and ongoing opioid use (mean percentage of opioid-positive urine screens: methadone 20.1, buprenorphine 12.5, *P*<0.001). Finally, the finding that employment and marital status was associated with a positive impact on psychological symptoms (i.e. lower symptoms) highlights the importance of supporting patients to achieve improvements in social functioning during treatment.

### Strengths and limitations

This study is strengthened by its large sample size and multisite design, which lends increased confidence in the results. The use of the MAP, a validated tool for assessment of symptoms in patients with substance use disorders, is an added strength. This study, like others, was susceptible to healthy user and volunteer biases, such that individuals with fewer psychological symptoms may have been more likely to participate. We have no way to explore this issue empirically. As is the case in all observational studies, we are unable to establish a causal relationship; whether a greater burden of psychological symptoms leads to increased substance use, or increased substance use causes a greater burden of psychological symptoms is unknown. The measurement of psychological symptoms in this study using the MAP^20^ (derived from the Brief Symptom Inventory,^21^ itself derived from the Symptom Check List 90^22^) does not capture the complete psychological profile of respondents, but rather focuses on anxiety and depression symptoms – a limitation of this study. However, the ability of the psychological symptoms subscale to discriminate between patients with psychiatric comorbidity as diagnosed by the MINI (higher psychological symptom scores) and those without psychiatric comorbidity (lower psychological symptom scores) as evidenced by a large effect size (Cohen's *d* = −1.01, 95% CI −1.18, −0.85) suggests that despite limitations, the measure discriminates between these groups of patients well.

This study is also limited by the assessment of psychological symptoms cross-sectionally. We note that the median duration in treatment was 2 years; whether these results generalise to patients newly starting MAT is unknown. We would hypothesise that patients newly starting MAT may be at greater risk of experiencing psychological symptoms in the context of possible opioid withdrawal, relapses to substance use or undertreatment of comorbid psychiatric disorders in the initial stages of MAT. In contrast to this hypothesis, we found that longer time in treatment was associated with slightly higher report of psychological symptoms.

How well these results would generalise to settings outside of Ontario, Canada, to other settings in North America or to other areas of the world is unclear. In Canada, MAT primarily takes on a harm-reduction role, such that retention in treatment is not contingent on abstinence from opioid or other substance use. In this setting, concerns around patient factors that may be associated with ongoing opioid or polysubstance use is inherently heightened, particularly because of risks of opioid overdose and mortality.

This study focused solely on one dimension of treatment outcome, namely substance use, as measured by urine drug screens. There are many other potentially important treatment outcomes that should be considered but fell outside of the scope of this study, including treatment retention, suicide-related behaviours, opioid overdose or death and the use of psychiatric or emergency department services.

### Directions for future study

Considering the notable prevalence and risks associated with suicidal ideation in this population, future research should explore the association between suicidal ideation and overdose, death and contact with psychiatric services. Whether different forms of MAT produce different outcomes with respect to psychological symptoms should also be investigated. Finally, future studies may wish to examine psychological symptoms over time and identify factors that influence their trajectory.

## Data Availability

The authors have ongoing access to all study data.
